# One-Step Electrodeposition Synthesized Aunps/Mxene/ERGO for Selectivity Nitrite Sensing

**DOI:** 10.3390/nano11081892

**Published:** 2021-07-23

**Authors:** Tan Wang, Cong Wang, Xianbao Xu, Zhen Li, Daoliang Li

**Affiliations:** 1 National Innovation Center for Digital Fishery, China Agricultural University, Beijing 100083, China; Tan.wang@cau.edu.cn (T.W.); ndfic_wc@cau.edu.cn (C.W.); xianbao_xu@cau.edu.cn (X.X.); leezn2009@163.com (Z.L.); 2 College of Information and Electrical Engineering, China Agricultural University, Beijing 100083, China; 3 China-EU Center for Information and Communication Technologies in Agriculture, China Agricultural University, Beijing 100083, China; 4 Beijing Engineering and Technology Research Center for Internet of Things in Agriculture, China Agricultural University, Beijing 100083, China; 5 Key Laboratory of Agricultural Information Acquisition Technology, Ministry of Agriculture, Beijing 100083, China

**Keywords:** electrochemical, nitrite sensor, AuNPs, Ti_3_C_2_-MXene, reduced graphene oxide, nanohybrid, modified electrode

## Abstract

In this paper, a new nanocomposite AuNPs/MXene/ERGO was prepared for sensitive electrochemical detection of nitrite. The nanocomposite was prepared by a facile one-step electrodeposition, HAuCl4, GO and MXene mixed in PBS solution with the applied potential of −1.4 V for 600 s. The modified material was characterized by scanning electron microscopy (SEM), energy-dispersive X-ray spectroscopy (EDS), X-ray diffraction (XRD) and cyclic voltammetry (CV). The electrochemical behavior of nitrite at the modified electrode was performed by CV and chronoamperometry. The AuNPs/MXene/ERGO/GCE showed a well-defined oxidation peak for nitrite at +0.83 V (Vs. Ag/AgCl) in 0.1 M phosphate buffer solution (pH 7). The amperometric responses indicated the sensor had linear ranges of 0.5 to 80 μM and 80 to 780 μM with the LOD (0.15 μM and 0.015 μM) and sensitivity (340.14 and 977.89 μA mM^−1^ cm^−2^), respectively. Moreover, the fabricated sensor also showed good selectivity, repeatability, and long-term stability with satisfactory recoveries for a real sample. We also propose the work that needs to be done in the future for material improvements in the conclusion.

## 1. Introduction

The demand of human beings for fish will reach 102 million tons by 2025, and aquaculture is one of the solutions to meet this projected requirement. However, the excretory products of fish, uneaten feed and overcrowding will cause the increase of nitrite concentration in aquaculture [1,2,3,4], which will affect fish growth [5], gill osmoregulatory function [6], blood oxygen carrying capacity [7,8] and ionic homeostasis [9]. Therefore, it is very important to detect nitrite content in aquaculture water rapidly and sensitively.

Many methods have been reported for nitrite determination, including Raman spectroscopy [10], spectrophotometry [11], chromatography [12], fluorescence analysis [13] and chemiluminescence [14]. Although all the aforementioned methods have shown excellent sensitivity and high accuracy, they need sophisticated instrumentation and complicated extraction processes, which are not appropriate for the rapid and real-time detection of nitrite. Electrochemical techniques [15,16] have the advantages of low cost, rapid, simple operation, and high efficiency [17]. However, the bare electrode has lower sensitivity and poor selectivity [18]. Modified electrodes with suitable catalysts are an effective solution for improve the selectivity and sensitivity [19], which have been widely used in nitrite detection.

Many electrochemical nitrite sensors were developed based on various nanocomposites that modify the electrode’s surface. C@In_2_O_3_ modified electrodes shows a linear range from 5 to 240 μM with a LOD of 0.08 μM for nitrite detection [20]. Muthumariappan et al. [21] developed a nitrite sensor based on a GO/Mn_3_O_4_ micro cubes modified screen-printed electrode, with a sensor performance of 0.1–1300 μM linear range with a 20 nM detection limit. Mani et al. [22] fabricated core-shell heterostructure multiwalled carbon nanotubes and reduced graphene oxide nanoribbons (MWCNTs@rGONRs)/chitosan nanocomposite for nitrite determination with a LOD of 10 nM, and the sensor’s performance was also good in actual samples. Jeena et al. [23] proposed an electrochemical nitrite sensor based on M-molybdate modified GCE with a linear range of 0.9–325.2 μM, the fabricated sensor that also can detect hydroquinone and Hg^2+^. These reports prove that a nanomaterial-modified electrode is effective for the detection of nitrite.

In addition, there are more nanomaterials can be used to improve the performance of the electrochemical sensor. Metal nanoparticles have large surface-to-volume ratios, which can increase electrochemical activities [24]. Commonly used metal nanoparticles include gold (Au) [25,26], silver (Ag) [27], platinum (Pt) [28,29], nickel (Ni) [30], palladium (Pd) [31], and copper (Cu) [32], the size, shape, composition, crystallinity and structure of metal nanoparticles affects their properties. Among them, Au nanoparticles (AuNPs) show excellent catalysis, electroconductibility and biocompatibility, which was widely used to fabricate the electrode to develop electrochemical sensors [33,34].

MXenes are a new 2D nanomaterial of 2D transition metal carbides, nitrides or carbonitrides developed by Prof. Gogotsi and co-workers in 2011, which have layered morphology, high electrical conductivities, high surface area, excellent hydrophilicity, good thermal stability, and environmentally friendly characteristics [35], and which have been used in electrochemical sensors [36], such as nitrite [37], pesticide carbendazim [38].

Graphene is a two-dimensional material composed of sp^2^ bonded carbon atoms, which has very large surface-to-volume (2630 m^2^/g), excellent electron transfer performance and biocompatibility [39,40]. The stability arising from long-range delocalisation of p-electrons giving rise to an extended aromatic network of C=C bonds across the entire basal plane. Recently, graphene-based material shows good electrochemical properties by the interactions of electroactive materials [16]. Some have reported that RGO shows significantly enhanced sensing performance compared to pristine graphene [41] and GO [42], due to it has more active edge plane defects and larger effective surface area. The electrochemical reduction method of GO to RGO is advantageous over chemical reduction methods due to its low cost, toxic free, green and fast approach [42]. The electrochemical sensors based on ERGO nanohybrid have been widely reported, such as in dopamine [43], nitrite [44] and Cd(II) [45] detection.

In this work, we have successfully prepared disposable AuNPs/MXene/ERGO/GCE composite by a one-step electrodeposition method. The AuNPs are uniformly loaded on the surface of MXene/ERGO. Due to the synergy of these components, AuNPs/MXene/ERGO show enhanced catalysis for the electrocatalytic oxidation of nitrite than those of ERGO, or MXENE/ERGO and AuNPs/ERGO. The modified electrode shows a good linear range of 0.5–80 and 80–780, and the stability, repeatability and selectivity of the modified electrode are also very satisfactory.

## 2. Materials and Methods

### 2.1. Chemicals and Apparatus

Graphite powder (99.95%), nitrite powder (99.8%), HAuCl_4_ (50%), Na_2_HPO_4_ (99.99%), NaH_2_PO_4_ (99.9%), KNO_3_ (AR, 98.5%), CaCl_2_(AR, 96%), NH_4_Cl (AR, 99.5%), KCl (AR, 99.5%), K_2_SO_4_ (AR, 99%) and glucose (99%) were purchased from Macklin Chemical Reagent Company (Shanghai, China). H_2_O_2_ (30%), HF (AR, 40%), HNO_3_ (68%), H_2_SO_4_ (98%), HPO_4_ (85%) and absolute ethanol (99.5%) was purchased from China Agricultural University, Beijing 100083, China. All of the chemical reagents were analytical grade and were used without further purification. Ultrapure water (18.25 MΩ) was used throughout the experiment and prepared using the laboratory equipment.

The glass carbon electrode (GCE) was obtained from Aida Heng-sheng Technology Development Co., LTD (AiDa, Tianjin, China). Cyclic voltammograms (CVs) (Gamry 600+, Warminster, PA, USA) and amperometric (i-t) (Gamry 600+, Warminster, PA, USA) measurements were performed by using the Gamry 600+ (Warminster, PA, USA) electrochemical workstation. The surface morphology and elemental distribution of the as-prepared AuNPs/MXene/ERGO/GCE was inspected by using the Hitachi SU3500 scanning electron microscopy (SEM) (Hitachi SU3500, Tokyo, Japan), X-ray diffraction (XRD) spectra were registered on a Bruker D8 diffractometer (Karlsruhe, Germany). The conventional three-electrode system was used for the electrochemical experiments; the modified GCE was used as a working electrode, a saturated Ag/AgCl as a reference electrode and a platinum wire as the auxiliary electrode. All the electrochemical experiments were performed at room temperature.

### 2.2. Preparation of Ti_3_C_2_-MXene/GO 

Graphite oxide prepared by Hummer’s Method [46], and 10 mg GO power was suspended in 10 mL deionized water and exfoliated through ultrasonication (2 h) to obtain graphene oxide (GO) solution (1 mg/mL).

We added 2.0 g of Ti_3_AlC_2_ powder gradually into 30 mL HF solution (50 wt %). The reaction was left to proceed for 48 h at 40 °C under continuous stirring. The resulting mixture was washed with distilled water for multiple cycles by centrifugation until the pH of the aqueous reached about 6.0, and then we obtained the final Ti_3_C_2_T_x_ power through vacuum-filtering.

We added 5 mL deionized water and 5 mL GO solution (1 mg/mL) to 5 mg Ti_3_C_2_T_x_ power respectively, and used ultrasonication (2 h) to obtainTi_3_C_2_T_x_ (MXene) solution (1 mg/mL) and GO/MXene solution (1 mg/mL).

### 2.3. Preparation of AuNPs/MXene/ERGO Modified Glass Carbon Electrode (GCE) 

The bare GCE was polished with 0.3 μm and 0.05 μm alumina slurries, and washed with HNO_3_, absolute ethanol and deionized water, respectively. We coated 5 μL GO, MXene and GO/MXene solution on the bare electrode surface and dried it at room temperature to obtain GO/GCE, Mxene/GCE and /MXene/GCE. 

**Scheme 1:**
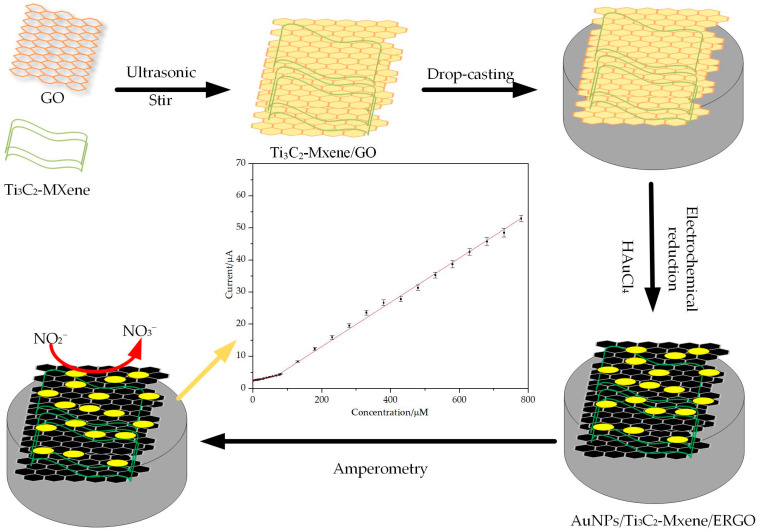
Nitrite detection scheme using ternary AuNPs/Ti_3_C_2_-MXene/ERGO modified glass carbon electrode (GCE) sensor.

The AuNPs/MXene/ERGO GCE was prepared was through a single electrodeposition. The GO/MXene/GCE immersed into the solution containing 5mM of HAuCl_4_ as source of Au^3+^ ion and 0.1 M PBS (pH 5) as electrolyte, and reduced at a potential of −1.4 V for 600 s to obtain AuNPs/MXene/ERGO/GCE. For comparison, the AuNPs/ERGO/GCE, and Au/MXene/GCE were also fabricated by the same method with the first preparation of GO/GCE and MXene/GCE. The ERGO/GCE was preparation by electrochemical reduction of GO at −1.4 V for 600 s 0.1 M PBS (pH 5) solution. Scheme 1 illustrates the preparation of AuNPs/MXene/ERGO/GCE.

### 2.4. Electrochemical Tests

The electrochemical tests were performed using a standard three-electrode system of electrochemical workstation, the GCE as the work electrode, platinum wire as the counter electrode and Ag/AgCl as the reference electrode. Cyclic voltammetry (CV) was conducted over a potential range of 0.4∼1.2 V at the scan rate of 50 mV s^−1^. Chronoamperometry was performed under the potential of +0.83 V. 

## 3. Results

### 3.1. Characterization of AuNPs/MXene/ERGO Nanocomposite

#### 3.1.1. Structural Characterization

**Figure 1:**
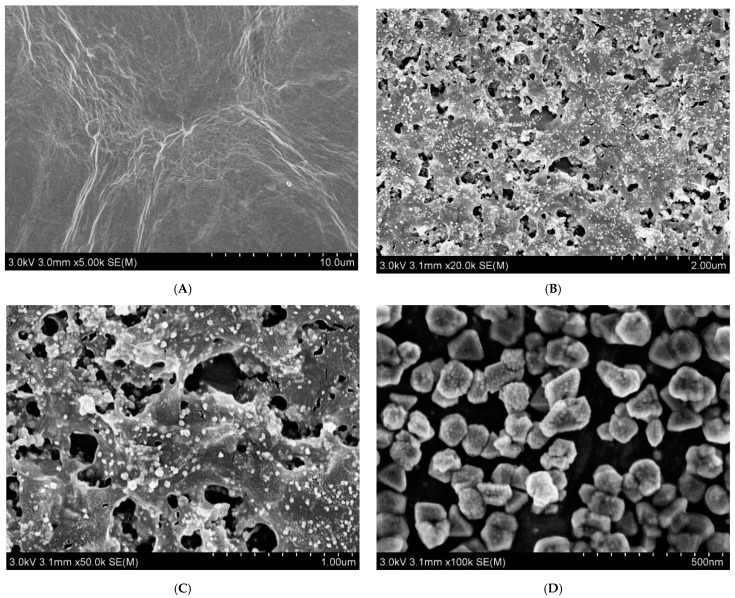
Scanning electron microscope (SEM) images of ERGO (**A**) and AuNPs/MXene/ERGO nanocomposite (**B**–**D**).

**Figure 2:**
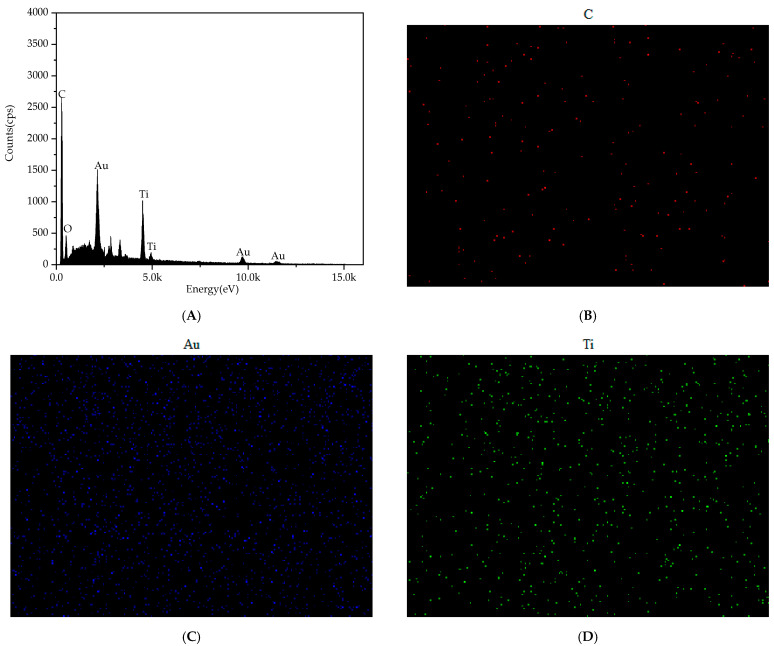
The energy-dispersive X-ray spectroscopy (EDS) (**A**) and element mapping of AuNPs/MXene/ERGO, (**B**) C. (**C**) Au and (**D**) Ti.

The morphology of ERGO and AuNPs/MXene/ERGO samples were investigated using SEM, as shown in Figure 1. Figure 1A shows the electrochemical reduction graphene oxygen, which displays the typical wrinkle-like thin sheet morphology. Figure 1B show the morphology of AuNPs/MXene/ERGO hybrid, it can clearly see that there are many porous on the surface, forming a three-dimensional structure. This is due to the addition of MXene, and the morphology of MXene changed during the electrochemical reduction process. In addition, it also shows that gold nanoparticles (AuNPs) are embedded on the surface of the material evenly, and the enlarged morphology (Figure 1C), reveals AuNPs also exist in the pores. The enlarged Figure 1D shows that the size of gold nanoparticles is uniform, and the particle size is about 100 nm, which is due to the prevention of agglomeration of AuNPs on the surface of MXene and ERGO. From SEM images, the composite material has a porous structure and the gold nanoparticles are loaded on the surface of ERGO and MXene. This structure is due to the fact that Mxene and GO are two-dimensional structures that favor the loading of AuNPs, and the mixture between Mxene and GO is preferentially drip-coated on the electrode surface. In the electrodeposition process, the Au^3+^ on the electrode surface are uniformly reduced to AuNPs, and the GO is also reduced to ERGO in this process. The elemental distribution of the nanocomposites was further determined by the energy-dispersed spectrum (EDS) show in Figure 2A and the two-dimensional elemental mapping described in Figure 2B–D, it clearly confirms the presence of C, Au, Ti, and O elements, illustrating the successful distribution of the target material.

**Figure 3:**
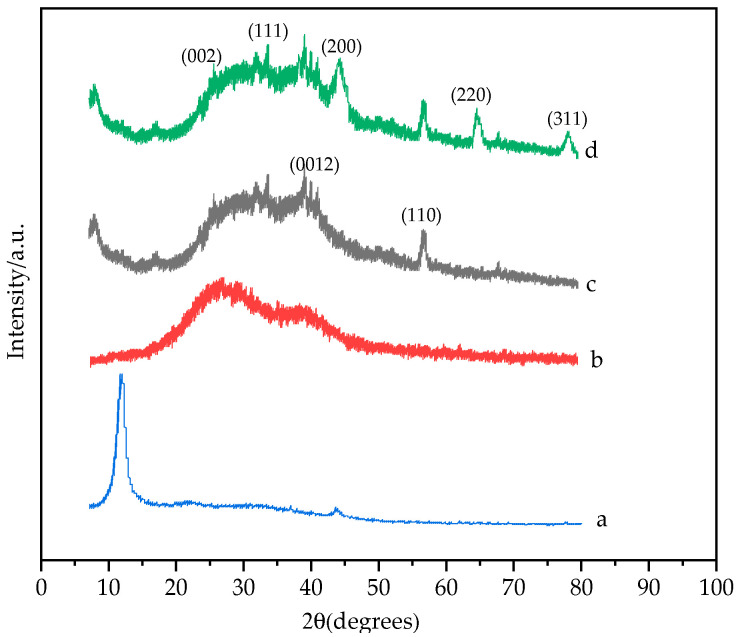
X-ray diffraction (XRD) patterns of GO (a), ERGO (b), MXene/ERGO (c) and AuNPs/MXene/ERGO (d).

Figure 3 shows the XRD patterns of GO (curve a), RRGO (curve b), MXene/ERGO (curve c) and AuNPs/MXene/ERGO (curve d). For curve a, the peak at 2θ = 11.2° corresponded to the (002) diffraction peak of GO, curve b shows the peak at 2θ = 27.8° indicating GO was reduced to ERGO after electrochemical reduction. From curve c, the typical characteristic peaks associated with the (0012) and (110) due to MXene were fitted well with Ti_3_C_2_ [38]. From curve d, the four sharp diffraction peaks occurring at 38.11°, 44.52°, 64.56°, and 78.14°, can be indexed to the crystal plane of (111), (200), (220), and (311) respectively, being in line with face-centered cubic Au. These results from SEM, EDS, XRD and the electrochemical results in the next section confirmed that an AuNPs/MXene/ERGO hybrid was successfully prepared.

#### 3.1.2. Electrochemical Characterization of AuNPs/MXene/ERGO Nanocomposite

**Figure 4:**
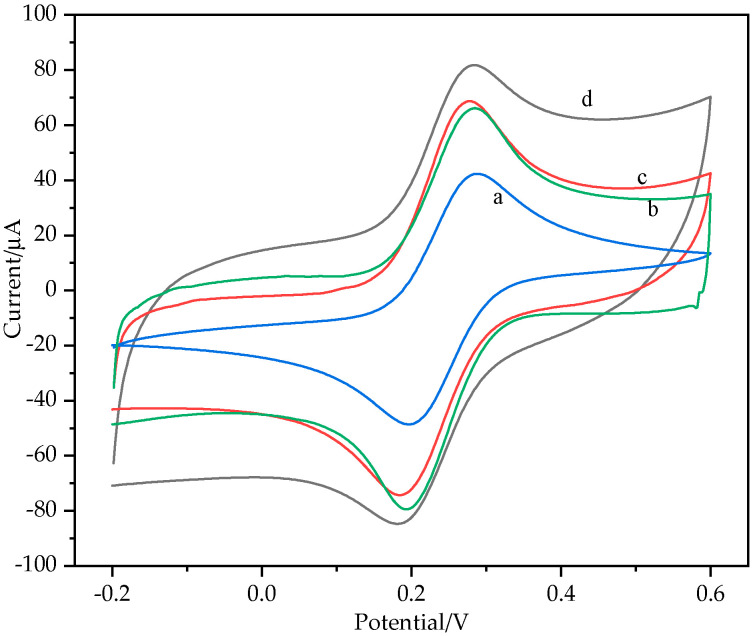
Cyclic voltammetry (CV) curves of Bare GCE (a), ERGO/GCE (b), MXene/ERGO/GCE (c), and AuNPs/MXene/ERGO/GCE (d) in 0.1 M KCl solution containing 5 mM [K_3_Fe(CN)_6_].

To study the effect of AuNPs/MXene/ERGO/GCE on the electron transfer reaction in the presence of the redox probe, CV was performed in 0.1M KCl solution containing 5 mM [Fe(CN)_6_]^3−^. The CV curves of bare GCE (a), ERGO/GCE (b), MXene/ERGO/GCE (c) and AuNPs/ERGO/GCE (d) shown in Figure 4. The peak current of bare GCE was 42.38 μA, ERGO/GCE and MXene/ERGO/GCE have higher peak currents, and it is shown that ERGO and MXene have the ability to enhance the oxidation reduction. Among them, AuNPs/MXene/ERGO/GCE obtained the highest oxidation peak current (81.83 μA). This indicates that the synergistic effect of AuNPs, MXene and ERGO increases the electron transfer rate of the modified electrode.

#### 3.1.3. Electrical Behavior of Modified Electrode toward Nitrite

**Figure 5:**
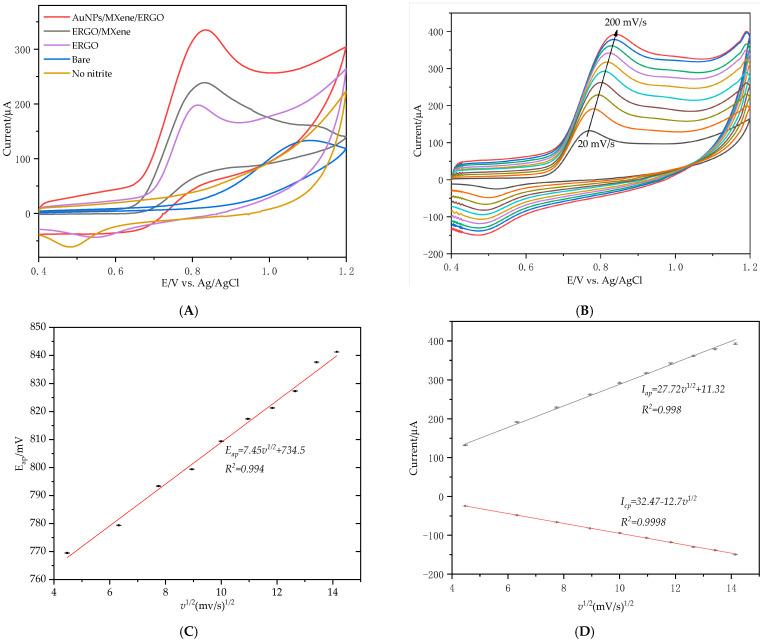
(**A**) CV result for Bare GCE, ERGO/GCE, ERGO/MXene/GCE and AuNPs/MXene/ERGO/GCE in 0.1 M PBS (pH 7) containing 5 mM NaNO_2_ with a scan rate of 50 mV/s, no nitrite is CV result for AuNPs/MXene/ERGO/GCE in 0.1 M PBS (pH 7) without nitrite with a scan rate of 50 mV/s. (**B**) CVs of AuNPs/MXene/ERGO/GCE in 0.1 M PBS (pH 7) containing 3 mM NaNO_2_ at different scan rates(*v*) from 20 to 200 mV/s (**C**) the relationship of anodic peak potentials vs. *v^1/2^*. (**D**) the relationship of current peak vs. *v^1/2^.*

To test the electrocatalytic oxidation effect of different modified electrodes on nitrite, CV curves of different modified electrode in 0.1 M PBS (pH 7) containing 5 mM nitrite with a scan rate of 50 mV/s show in Figure 5A, each group was measured five times. The result shows that the nitrite oxidation peak and anodic peak potential of ERGO/GCE, ERGO/MXene/GCE and AuNPs/MXene/ERGO/GCE are higher than bare GCE, bare GCE shows higher oxidation potential of 1.1 V and lower oxidation peak current; ERGO/GCE with the oxidation peak potential of +0.81 V, and the oxidation peak current is 1.4-fold that of bare GCE, and this is due to the larger specific surface area and excellent electrical conductivity of ERGO. The oxidation peak current of ERGO/MXene/GCE is higher than ERGO/GCE, and the reason for this is that both ERGO and MXene have large specific surface areas and faster electron transfer rates. Among them, AuNPs/MXene/ERGO/GCE shows a well-defined, enhanced nitrite oxidation peak, it was about 2.5-fold that of bare GCE, this is the result of synergistic effect between ERGO, MXene and AuNPs. The CV curve of no nitrite is AuNPs/MXene/ERGO/GCE at 0.1M PBS without nitrite, there was an no oxidation peak without add nitrite, indicating that AuNPs/MXene/ERGO/GCE had the oxidation peak of nitrite at +0.83 V. 

To understand the electrochemical mechanism, CV curves of 3 mM NO_2_^−^ in PBS (pH = 7) were recorded AuNPs/MXene/ERGO/GCE at different scan rates from 20 and 200 mV/s (Figure 5B). The result showed that the anodic peak currents (I_ap_) of AuNPs/MXene/ERGO/GCE increased accordingly with the increase of the scan rate, meanwhile the cathodic and anodic peak potentials show a small shift and the peak-to-peak separation also becomes slightly enlarged, Figure 5C shows that the anodic peak potential shift is related to the scanning rate, the relationship of E_ap_ vs. *v^1/2^* with a correlation coefficient of 0.994(R^2^). Figure 5D indicates a good linear relationship of I_ap_ and I_cp_ vs. *v^1/2^* from 20 and 200 mV/s (linear regression equations: *I*_ap_ = 11.32 + 27.72*v^1/2^*, R^2^ = 0.998; *I*_cp_ = 32.47-12.7*v^1/2^*, R^2^ = 0.9998). These results reveal that the electrochemical oxidation of nitrite at AuNPs/MXene/ERGO/GCE is a typical diffusion-controlled process. Overall oxidation of nitrite response at AuNPs/MXene/ERGO/GCE follows the following mechanism as shown Equation (1):

NO_2_^−^ + H_2_O → NO_3_^−^ + 2H^+^ + 2*e*^−^ (1)

### 3.2. Effect of Solution pH

**Figure 6:**
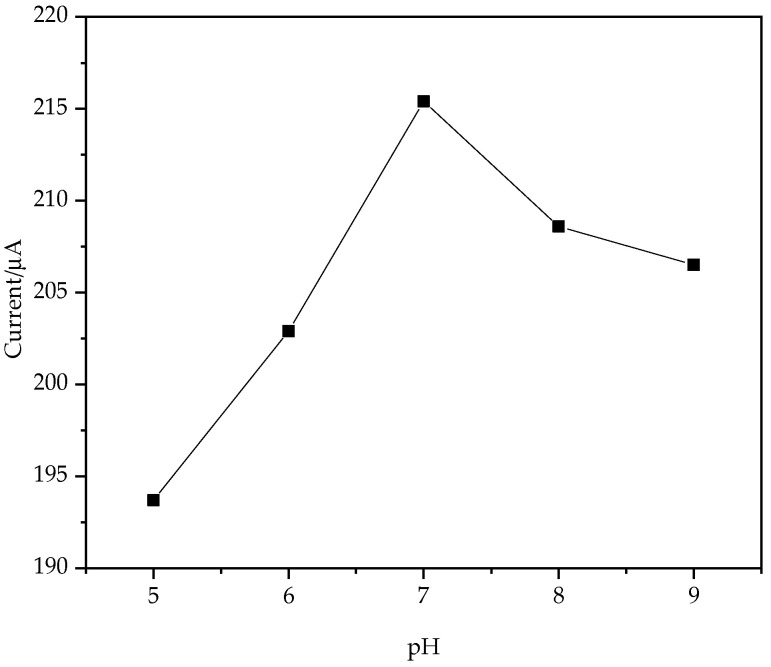
CV of AuNPs/MXene/ERGO/GCE in 0.1 M PBS containing 3 mM nitrite at different pH values (pH = 5, 6, 7, 8, 9), scan rate = 50 mV/s.

The pH value has great influence on the electrochemical performance of the modified electrode. Here, the electrochemical performance of AuNPs/MXene/ERGO/GCE was studied towards 3 mM nitrite by CV in the pH range of 5 to 9 in 0.1 M PBS, the current peak as depicted in Figure 6. It can be seen that the current response is the strongest when the pH values are at 7, the current response increase when the pH lower than 7, and then declined significantly when the pH values are higher than 7. Consequently, the pH 7 of 0.1 M PBS solution was selected as a support electrolyte solution for the sensitivity detection of nitrite.

### 3.3. Amperometry

**Figure 7:**
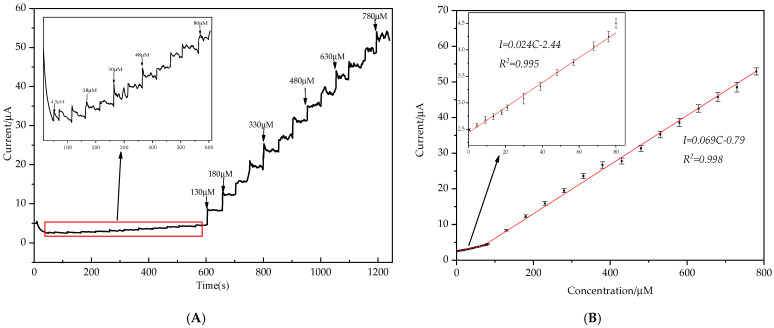
(**A**) Amperometric responses of AuNPs/MXene/ERGO/GCE in continuous add of NO2^−^ into 0.1 M PBS (pH 7) at an applied potential of 0.83 V (with stirred: 1200 r/min). Inset is the enlarge amperometric responses in a low concentration of nitrite. (**B**) Plot of relationship of current with nitrite concentration. Inset is the enlarge part of calibration plot.

The modified GCE performance for the detection of nitrite of the AuNPs/MXene/ERGO/GCE was also investigated by amperometry. As shown in Figure 5A, the maximum response current with a good signal/noise ratio was observed at +0.83 V of AuNPs/MXene/ERGO/GCE. Therefore, +0.83 V was selected as the optimal detection potential in subsequent experiments. Figure 7A show the amperometric responses of modified electro at +0.83 V with adding nitrite in continuously stirred PBS (0.1 M, pH 7, 1200 r/min) solutions, and the nitrite addition interval was about 50 s. With the addition of nitrite, the response current increases with the increase of nitrite concentration. Fifty response current data of nitrite were obtained in 50 s, and the average value of these data was used as the response current of nitrite; they show a good linear relationship between the nitrite concentration and response current in Figure 7B. The linear ranges were from 0.5 to 80 μM and 80 to 780 μM with correlation coefficient of 0.995 and 0.998, respectively. The limit of detection (LOD) was 0.15 and 0.051 μM (LOD = 3SD/k), where SD and k are standard deviation of the blank and the slope of calibration graph, respectively. The limit of quantitation (LOQ) was 0.5 and 0.17 μM (LOD = 10SD/k), and the sensitivity was 340.14 and 977.89 μA mM^−1^ cm^−2^, respectively. Compared with others report nitrite sensors in Table 1, the proposed nitrite sensor shows similar performance in detection ranger and LOD but shows higher sensitivity. The results indicate that the fabricated AuNPs/MXene/ERGO/GCE could be an alternative platform for the detection of nitrite.

### 3.4. Selectivity, Reproducibility and Stability of the Modified Electrode

**Figure 8:**
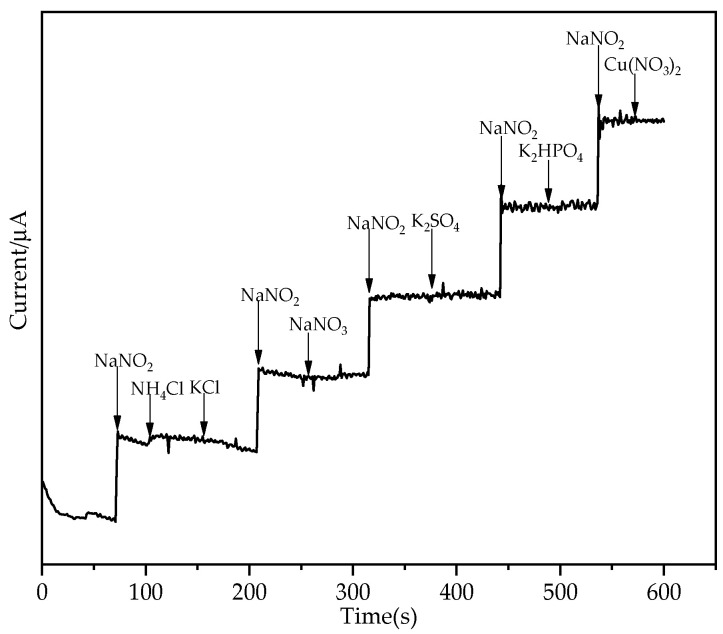
Typical amperometric current responses at AuNPs/MXene/ERGO/GCE for addition of 50 μM nitrite and each addition of interference species 2 mM NH_4_Cl, NaNO_3_, K_2_SO_4_, K_2_HPO_4_, 3 mM KCl, 500 μM Cu(NO_3_)_2_. Applied potential: 0.83 V.

The selectivity is an important parameter of the nitrite sensor. The selectivity of AuNPs/MXene/ERGO/GCE toward nitrite sensing requires the addition of various possible interferents in 0.1 M PBS (pH = 7.0) by the amperometric method. Figure 8 shows amperometric current response for 50 μM nitrite in presence of 2 mM NH_4_Cl, NaNO_3_, K_2_SO_4_, K_2_HPO_4_, 3 mM KCl and 500 μM Cu(NO_3_)_2_, it clear that these ions showed almost no interference, the presence of 40-fold NH_4_Cl has a slight effect on the current response. These results demonstrated the excellent selectivity of AuNPs/MXene/ERGO/GCE for the detection of nitrite.

**Figure 9:**
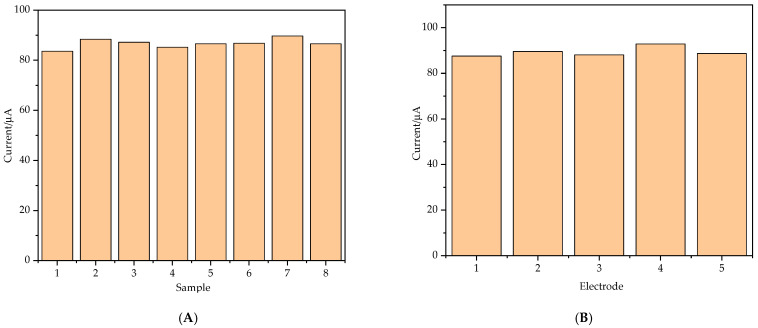
(**A**) AuNPs/MXene/ERGO/GCE detection nitrite in 8 samples with the same concentration. (**B**) The test of five AuNPs/MXene/ERGO/GCE in the same sample.

The parameters of stability, flexibility, and reproducibility of a fabricated sensor are very important in real and industrial applications. These parameters have been tested using cyclic voltammetry experiments of nitrite with a concentration of 1 mM. The relative standard deviation (R.S.D) was calculated as 2.35% from five different electrodes responses for detection of 1 mM nitrite (as show in Figure 9A), and was 2.13% for eight successive measurements (as show in Figure 9B), confirming the reproducibility of the sensor. The fabricated sensor kept in air tight packings, current peak was decreased 5.78% after two weeks toward detection of 500 μM nitrite. These results reveal that the fabricated sensor has a suitable operational and storage stability.

In order to evaluate the practical applicability of the fabricated sensor, nitrite present in tap water and river water were determined by amperometry using a standard addition method. The collect samples were filtered with a 0.22 μm membrane to remove large impurities. To all samples were added nitrite concentrations of 5, 10 and 15 μM, and all samples were tested three times to take the average value. Table 2 show the recoveries were: tap water (97.8%, 95.7% and 105.2%) and tap water (97.2%, 95% and 103.2%). The good recoveries of nitrite indicate that the developed nitrite sensor has a potential applicability for real water sample analysis.

## 4. Conclusions

In this work, we prepared a nitrite electrochemical sensor constructed with ERGO, AuNPs and an MXene composite modified electrode. The AuNPs were uniformly coated on the surface of MXene/ERGO by a simple electrodeposition. Under the optimal conditions, the AuNPS/MXene/ERGO/GCE exhibited a good current response compared to other electrode because of the synergistic effect between AuNPs and MXene, as well as ERGO supports, and the conductivity and catalytic performance of the electrode are improved. Compared with other reported sensors, the performance of the sensor was significantly improved. The amperometry experimental results demonstrated that the fabricated sensor had linear ranges (0.5 to 80 μM and 80 to 780 μM); low detection limit (0.15 and 0.51 μM), high sensitivity (340.14 and 977.89 μA mM^−1^ cm^−^^2^), and also obtained good selectivity, reproducibility, stability and satisfactory recovery. Therefore, the fabricated sensor has potential for the actual detection of nitrite.

However, there is still some work to be done in the practical application of the proposed sensor. In order to obtain better performance, the synthesis of modified electrode materials needs to be optimized. It is very important to optimize the mixing ratio of MXene and ERGO, and the amount of drop-casting on the electrode, and the electrodeposition voltage, time and pH value also affect the properties of the prepared materials. In addition, how to protect the electrode and increase the service time of the electrode are equally important.

## Figures and Tables

**Table 1:** Comparison of proposed sensor with another reported nitrite sensor.

Modified Electrodes	Linear Range (μM)	LOD (μM)	Ref.
NPCF-GNs/GCE	0.1–100	0.088	[47]
MOF-525/GNR/ITOE	10–2500	0.75	[48]
Graphene/GCE	1–250	0.24	[49]
GO-Ag/GCE	10–180	2.1	[50]
GO-CS-AuNPs/GCE	0.9–18.9	0.3	[51]
CR-GO/GCE	8.9–167	1	[52]
PDAB/ERGO/GCE	7–20,000	0.03	[53]
CoHCF/RGO/GCE	1–100	0.27	[54]
fZnO/rFGO/GCE	10–8000	33	[55]
Fe3O4/r-GO/GCE	1–92	0.3	[56]
AuNPs/MXene/ERGO/GCE	0.5–80	0.15	This work
	80–780	0.051	

**Table 2:** Determination of nitrite in water samples (n = 3).

Real Sample	Added (μM)	Found (μM)	Recovery (%)
Tap water	5	4.89	97.8%
	10	9.57	95.7%
	15	15.78	105.2%
River water	5	4.86	97.2%
	10	10.5	95%
	15	15.48	103.2%

## Data Availability

The data presented in this study are available upon request from the corresponding author.
